# Advances in Targeting Growth Factor Signalling in Neuroblastoma and Overcoming Drug Resistance

**DOI:** 10.3390/cells15010004

**Published:** 2025-12-19

**Authors:** Karina Ivanenko, Ruslan Shaymardanov, Vladimir Prassolov, Timofey Lebedev

**Affiliations:** 1 Engelhardt Institute of Molecular Biology, Russian Academy of Sciences, 119991 Moscow, Russia; karina.ivanenko@mail.ru (K.I.); prassolov45@mail.ru (V.P.); 2 Advanced Engineering School, ITMO University, 192001 Saint-Petersburg, Russia; ruslan.shaymardanov2001@mail.ru

**Keywords:** neuroblastoma, growth factors, receptor tyrosine kinase, drug resistance, AXL, heterogeneity

## Abstract

Neuroblastoma is an embryonal tumour that arises from the malignant transformation of neural crest cells and remains one of the deadliest malignancies in children under five. Neural crest development is regulated by dynamic switches in transcriptional programmes, guided by a variety of growth factors. Due to its developmental origin, neuroblastoma is unique in that these tumours often retain overactivation of growth factor signalling, which can be targeted by receptor tyrosine kinase (RTK) inhibitors. However, mutations in kinases, except for ALK, are extremely rare in neuroblastoma. Furthermore, the high degree of intratumoural heterogeneity often renders RTK inhibition ineffective as a monotherapy. For high-risk tumours, which lack effective treatment options, there remains an unmet need for targeted therapies. This review summarises the roles of growth factor receptors in neural crest and neuroblastoma development in light of recent single-cell studies. We provide a systematic overview of RTK inhibitors that can target growth factor signalling in neuroblastoma and detail their current status in clinical development. We also explore the role of intratumoural heterogeneity in resistance to RTK inhibitors, focusing on the adrenergic-to-mesenchymal transition, which drives a switch in growth factor receptor expression. Finally, we discuss strategies to overcome RTK inhibitor resistance by targeting neuroblastoma cell plasticity, disrupting downstream signalling pathways, or inhibiting escape mechanisms from cell death. This review provides a theoretical basis for developing novel combination therapies incorporating RTK inhibitors.

## 1. Introduction

Neuroblastoma (NB) is a malignant tumour that arises from the sympathetic nervous system during embryonic development [1]. It is the third most common childhood cancer, after acute lymphoblastic leukaemia and brain tumours, and accounts for about 8% of all paediatric malignancies. NBs cause approximately 15% of childhood deaths from cancer, while the survival rate for NB varies widely, ranging from 5% for more aggressive tumours to nearly 100% for those with a favourable prognosis [2,3,4,5,6,7,8,9,10]. Still, therapeutic options for high-risk patients remain extremely limited, and there is an unmet need for the development of novel targeted therapies for NB. NB tumours arise from neural crest cells that undergo a complex differentiation process. Growth factors, whose receptors primarily belong to receptor tyrosine kinase (RTK) family, play a crucial role in governing neural crest differentiation, proliferation, and migration at different stages. NB cells often retain some of the stem cell properties from their neural crest progenitors, including the high expression of growth factor receptors and other RTKs [11,12,13], which are usually lost during late differentiation. This gives a therapeutic opportunity to target growth factor receptors expressed by NB cells with RTK inhibitors (RTKi), which were already approved for the treatment of other cancers.

RTKi revolutionised the treatment of BCR-ABL1-positive chronic myeloid leukaemia, human epidermal growth factor receptor 2 (HER2)-positive breast cancer, and epidermal growth factor receptor (EGFR)-mutant non-small cell lung cancer (NSCLC) [14]. Despite more than a hundred U.S. Food and Drug Administration (FDA)-approved RTKi and several hundred in late development stages, none of them are approved for treatment of NB, and they are only used as experimental drugs. However, unlike most cancers, where activating mutations in one or several RTKs are common, NB has a very low rate of point mutations. The only recurrent RTK mutations in NB are ALK mutations, which appear in only 7–10% of NB tumours. Still, numerous RTKi were shown to be effective against cancer cells without activating mutations in RTKs if they have overactivation of those RTKs or are addicted to RTK signalling. However, rapid development of resistance remains one of the main challenges in the use of RTKi, as growth factors and their receptors often initiate the same signalling pathways and can substitute each other. In this review we summarise data on growth factor signalling and its impact on NB malignant transformation and focus on the existing inhibitors which are used or could be potentially used for NB treatment. We also overview the role of growth factor signalling in neural crest development, which is essential to an understanding of NB development, in the light of recent single-cell studies [15,16,17,18].

## 2. Methods

### Analysis of Drug Screening and Gene Fitness Data

Gene dependency scores were obtained from DepMap database (https://depmap.org/portal/ (accessed on 10 October 2025) 24Q2 version [19], using post-Chronos gene effect values and DEMETER2 Data v6 scores, and scores were combined as previously described [20]. According to DepMap [21], genes with scores less than −1 are essential to cell survival, and depletion of genes with scores less than −0.5 strongly inhibits cell survival. Drug response data was obtained from PRISM primary repurposing dataset 24Q2, where drug effects on cell lines were screened at single 2.5 µM dose. NB cell lines were selected according to DepMap “OncotreePrimaryDisease” metadata classification. We calculated median drug responses for each cancer type, according to DepMap metadata classification, and then calculated z-scores for median values. We also compared drug responses between NB cell lines and all other cancer cell lines using the Mann–Whitney non-parametric test. Drugs with z-score values < −1 for NB cells and *p* < 0.05 for comparison of NB to all other cell lines were considered as selective for NB. Gene dependency scores were compared in the same manner. Z-scores, median values, and *p*-values are provided in Table S1.

## 3. Growth Factors in Neural Crest Development

**Figure 1:**
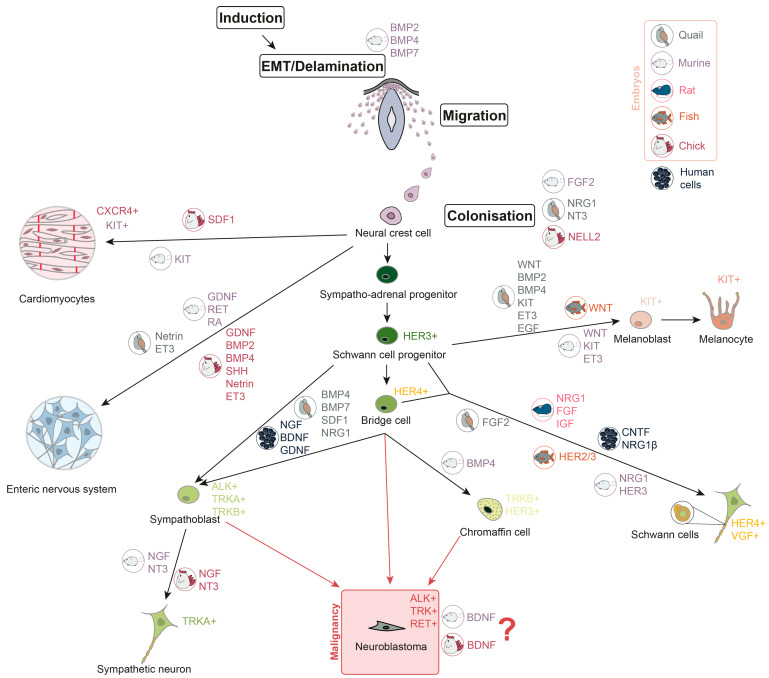
Neuroblastoma development during neural crest cells differentiation. Growth factors, involved in different stages of neural crest cells differentiation, are shown in colours, representing the embryonic origin in which their involvement is confirmed (grey represents quail embryos, mauve—murine, pink—rat, orange—fish, red—chick, and black—human cells). Receptor tyrosine kinases, which are characteristic of differentiated cells, are also listed in the picture.

Neural crest cells (NCC) are multipotent and actively migrate, giving rise to various tissue precursors, including those that form the peripheral and enteric nervous systems, melanocytes, Schwann cells, chromaffin cells, and cardiac progenitor cells (Figure 1). After migrating from the neural crest, the cells form sympathetic ganglia, the adrenal medulla, and paraganglia [22,23]. At various stages of development, NCC respond to a variety of growth factors that determine their growth and direction of migration. Overall, NCC proliferation and differentiation are mostly controlled by four signalling pathways: Wingless/Int (WNT), fibroblast growth factor (FGF), bone morphogenetic protein (BMP), and Notch [24]. WNT/β-catenin signalling is important for the formation of the peripheral nervous system. Activation of β-catenin promotes the formation of the sensory neurons in the dorsal root ganglia (DRG), whereas downregulation of WNT/β-catenin signalling reduces DRG and sympathetic ganglia development [25]. In vitro experiments show that human neural crest stem cells (hNCSC) could be differentiated into peripheral sympathetic neurons and sensory neurons by exposure to brain-derived neurotrophic factor (BDNF), glial cell line-derived neurotrophic factor (GDNF), nerve growth factor (NGF), and dibutyryl cyclic AMP (dbcAMP) [26]. As for chromaffin cells, it is still largely unknown which factors are involved in their development. As far as it is investigated for chicks and mice, BMP-4 may play a role in chromaffin cells’ maturation, but not differentiation [27]. However, in human adrenal gland chromaffin cell precursors express progenitor and migratory markers such as neurotrophic receptor tyrosine kinase 2 (*NTRK2*) and *ERBB3* (which encodes HER3) [15]. GDNF and its receptor rearranged during transfection (RET), retinoic acid (RA), are crucial for the development of murine enteric nervous system [28,29,30], and GDNF, BMP2, BMP4, sonic hedgehog (SHH), netrin, endothelin-3 (ET3), and their receptors are required for the development of avian enteric nervous system [31,32,33,34,35]. Melanoblast specification in neural crest is controlled by WNT signalling [36,37,38], BMPs [37,39], KIT [40,41], ET3 [42,43,44], and epidermal growth factor (EGF) [45]. Migration of the C-X-C chemokine receptor 4 (CXCR4)-expressing cardiac NCC in chick embryos depends on stromal-derived factor-1 (SDF1), which works as a chemoattractant [46]. Also, it has been found that c-KIT+ cardiac progenitor lineage is able to generate cardiomyocytes in murine embryos [47]. Description of growth factor involvement in Schwann cell development was not summarised before, but novel single-cell analyses shed light on this. Schwann cell induction is conducted by the ciliary neurotrophic factor (CNTF), neuregulin 1β (NRG1β) and dbcAMP in hNCSC in vitro [26]. It was shown that NRG1 type III has a key role in Schwann cells’ maturation in mammalian (rat and murine) cells [48,49] and FGFR2 is involved in their differentiation in quail [45]. HER2/3 controls myelination by Schwann cells and migration of their precursors in fish. Meanwhile, HER3 is involved in regulation of Schwann cell precursor migration in mice [50]. According to the single-cell transcriptomic analyses, HER4-positive bridge cells, a transition state between progenitors and differentiated cells, connect Schwann cell progenitors (SCP) and chromaffin cells [18]. A combination of FGF and insulin-like growth factor (IGF) promotes survival of SCP in rat embryos [51]. BMP, SDF1, and NRG1 are the key factors to control the development of sympatho-adrenal cells in quails [52]. Epithelial–mesenchymal transition (EMT) and delamination stages of NCC development are regulated by BMPs (BMP2, BMP4, and BMP7) in mice, which is common for other mammals [16]. FGF2 in murine embryos, NRG1, neurotrophin-3 (NT3) in quail embryos, and neural epidermal growth factor-like 2 (NELL2) protein kinase in chick embryos were shown to promote NCC proliferation and differentiation [53,54,55].

In addition, some proteins are necessary for the formation of the nervous system as a whole. Neurotrophins (NT) are a group of proteins that play a crucial role in the development and maintenance of the nervous system. There are 4 neurotrophins: NGF, BDNF, NT3, and NT4. These proteins work together to ensure the proper functioning of neurons and other nerve cells [56]. NGF and NT3 are required for the formation of sympathetic neurons [57]. Receptor tyrosine kinase *KIT* is expressed by NCC and is necessary for their survival, proliferation, and migration [58,59]. KIT-positive cells also give rise to subpopulations of cardiomyocytes, neuronal, and glial cells [47,60]. FGF is important for NCC induction and specification. It inhibits BMP signalling and *BMP* expression, and induces WNT expression [61]. Loss of RET in mice leads to deprivation of enteric neurons in the digestive track [62]. Moreover, RET is important for the development of the nervous system overall [63].

It is still not clear from which specific subpopulation of neural crest progeny, and at which differentiation stages, NB arises. There are two main possible variants: sympatho-adrenal cells or chromaffin cells [16]. According to the single-nuclei transcriptome analysis, an undifferentiated cluster of cells in high-risk NB samples is similar to the progenitor population in postnatal human adrenal glands, as they share high expression of *NTRK2* and *ERBB3* [15]. Presence of mature sympathoblasts and late SCP is associated with favourable NB prognosis, while the proliferation of sympathoblasts and chromaffin cells is associated with unfavourable prognosis [17,18]. Moreover, a novel transitory state between chromaffin cells and sympathoblasts has been discovered, which may be a loophole for NB cells occurrence [17]. Also, it has been shown that the induction of BDNF in chick and murine sympathoblasts in vitro leads to cell proliferation, which may contribute to the NB development [57]. In addition, chromaffin cells are likewise considered to be a site of NB origin [64,65]. Involvement of growth factors in neural crest development and malignant transformation is summarised in Figure 1.

## 4. Growth Factors and Their Receptors in Neuroblastoma

Malignant transformation can occur during different stages of cell differentiation and may result in the expression of some stem cell or progenitor markers in tumour cells, including growth factor receptors, most of which belong to RTK family. Also, neuroblast cells can secrete certain growth factors, which often lead to the formation of autocrine loops that support the growth of malignant cells [66,67,68,69].

### 4.1. ALK

Mutations in *ALK* gene are considered as one of the drivers of NB development and progression [70], and ALK contribution to NB development has been thoroughly described [71,72]. ALK-positive NB cells are thought to arise mainly from sympathoadrenal lineage [18], and ALK mutations impair differentiation of those cells [73]. There are three main point mutations within the ALK tyrosine kinase domain—R1275, F1174, and F1245—which are found in familial and sporadic NB cases [74,75]. *ALK* focal genomic amplification is present in 2–10% of NBs; patients with this alteration have an unfavourable prognosis [76], while *ALK* translocation in NB has been reported only rarely [77]. ALK alterations are associated with poor prognosis and suppressing the expression or inhibiting the activity of ALK in cells with a mutant form or overexpression of *ALK* have been shown to reduce the cell and tumour growth rate [71,72]. The ALK ligand ALKAL2 can be secreted by adrenocortical-like stromal cells within NB tumours but not by cancer neuroblasts themselves [78]. This suggests that in tumours with high ALK expression but lacking ALK mutations, oncogenic signalling may be driven by microenvironmental ligands such as ALKAL2, potentially derived from the adrenal medulla. Tumours enriched for this adrenocortical-like signature are likely localised to the adrenal medulla and are associated with a significantly better patient survival probability [78].

### 4.2. TRK

The most studied signalling cascades, the activity of which affects NB development, are the ones associated with NTs [72]. There are two types of NT receptors: the tropomyosin receptor kinase (TRK) RTKs (TRKA, TRKB, and TRKC), and the NGF receptor (p75NTR), which belongs to the family of tumour necrosis factor receptors (TNF receptor superfamily) [79,80]. The TRKA, TRKB, and TRKC receptors have selectivity for certain NTs: NGF binds predominantly to TRKA, BDNF and NT4 to TRKB, while NT3 binds to TRKC [80]. Similar to ALK, TRKA is expressed on the sympathoadrenal lineage. NB tumours have higher TRKA expression than other cancers and the mature adrenal gland [12]. However, higher TRKA expression in NBs is generally associated with a favourable prognosis and, unlike ALK, does not seem to impair cell differentiation. The absence or presence of NGF in tumour microenvironment (TME) results in spontaneous regression or differentiation of NB cells, respectively [81]. Schwann cells are known to be the source of NGF or another NT. These cells can trigger immature NB cells to differentiate into benign ganglioneuroblastomas and ganglioneuroma [82].

The TRKA receptor is typically expressed in low-risk NBs that are prone to spontaneous regression [83], and TRKA’s role in NB regression and differentiation is discussed in detail in [71,79]. NGF-activated TRKA is connected to increased immunogenicity by overexpression of major histocompatibility complex I (MHC I) [84].

Although *NTRK1* (which encodes TRKA) expression is generally a beneficial factor, a shortened isoform, TRKAIII, has been identified as a product of alternative splicing, which is predominantly found in aggressive NB [85]. The shortened isoform of TRKAIII mRNA lacks exons 6, 7, and 9, which leads to the deletion of one of the immunoglobulin-like domains. As a result, TRKAIII isoform is activated even in the absence of NGF [86]. TRKAIII has been suggested to be potentially oncogenic, as NB cells with overexpressed TRKAIII tend to form more aggressive tumours in mice [87]. Although TRKA is considered a factor contributing to tumour regression, NGF can both protect NB cells from doxorubicin or enhance its action. RTKi also upregulate TRKA expression and NGF can compensate for other RTK inhibition by sustaining ERK activity [12].

Unlike *NTRK1*, *NTRK2* and its ligand *BDNF* are expressed in NBs with an unfavourable prognosis, and their expression correlates with the amplification of the *MYCN* gene [83,88]. Co-expression of the ligand and receptor leads to the formation of an autocrine loop, which can increase the survival of neuroblast cells and promote metastasis [66,67]. NB cells that survive chemotherapy in vitro produce more BDNF, possibly allowing them to survive through an autocrine loop [68]. Although TRKA and TRKB are both expressed on sympathoblasts and chromaffin cells, they seem to have different functions. TRKB expression in chromaffin cells is increased under hypoxia [89], which may also contribute to its pro-survival role in NB.

Much less is known about the role of TRKC and p75NTR in NB development. In general, increased expression of *NTRK3* (which encodes TRKC) or *NGFR* (which encodes p75NTR) is associated with less aggressive NBs. Suppression of NT3 expression or blocking of TRKC with antibodies in NB cell lines expressing NT3 and TRKC resulted in the induction of apoptosis and inhibition of xenograft tumour growth [90]. The low-affinity p75NTR is mainly expressed in ganglioneuroma and ganglioneuroblastoma neuroblast cells, as well as in differentiated NBs. Ectopic expression of *NGFR* in NB cell lines reduces the rate of cell proliferation, increases the number of apoptotic cells in vitro, and prevents tumour formation in vivo [91].

### 4.3. ERBB Family

The expression of *EGFR* was detected in both primary NB and in NB cell lines [92]. EGF stimulates NB cell proliferation in vitro, and EGFR inhibition by gefitinib induces apoptosis in NB cell lines [93]. EGFR and HER2 inhibitor, afatinib, blocks the growth of neuroblast cells in vitro and in vivo, and restores NB cell sensitivity to doxorubicin [94]. It has been shown that simultaneous inhibition of several receptors from the ERBB family leads to a more significant effect on NB growth in vivo [95]. Notably, EGF results in increased expression of *MYCN* through activation of the MAPK/ERK signalling cascade in NB cells [96]. Moreover, a recent single-cell study showed that heparin-binding EGF-like factor (HB-EGF) and HER4 receptor are involved in a crosstalk between neuroblasts and macrophages in human and mice tumours. HB-EGF secretion by macrophages derived from THP-1 cells increased HER4 and ERK phosphorylation in several NB cell lines, and co-culture with macrophages increased colony-formation potential on NB cells [97]. HB-EGF is secreted only by TME in NB samples and is connected with NB differentiation [98]. *EGFR*, *ERBB2* (which encodes HER2), and *ERBB4* (which encodes HER4) are highly expressed on NB cells with upregulated mesenchymal gene signature, which is associated with a subset of more resistant cells [18,99]. Although two recent studies assigned *ERBB4* expression to sympathoadrenal cells, and EGFR to mesenchymal [97,100], which warrants further studies on *ERBB4* expression in NB cell types. HER3 and HER4 are expressed on Schwann cell progenitors and bridge cells, which have high plasticity and closely resemble NB cells with a mesenchymal signature [101]. Given the high plasticity of bridge cells, it is possible that HER3- or HER4-positive cells transition between mesenchymal-like and sympathoadrenal-like cell states. *ERBB4* expression, along with *NTRK2,* was detected in NB cells migrating along nerves in an avian embryo [102], suggesting a possible major role of HER4 in NB progression.

### 4.4. FGFRs

N546K point mutation in *FGFR1* gene is the most frequent *FGFR1* gene alteration in cancer [103], and it was found in NB tumours as a clonal variant, suggesting its role as a driver mutation [104]. High levels of *FGFR1* mRNA correlate with low relapse-free survival; *FGFR1* silencing by shRNA inhibits cologenicity and invasion in SH-SY5Y and SK-N-BE2 cells, and overexpression of *FGFR1^N546K^* promotes cell invasion and colonigenicity [105]. The silencing of *FGFR2* leads to sensitisation to cisplatin of cisplatin-resistant NB cells [106]. RNA sequencing of NB cells resistant to lorlatinib (ALK inhibitor) shows that *FGFR2* is expressed at a higher level compared to the parental ones [107]. Also, *FGFR2* expression is associated with unfavourable prognosis [108] and expression analysis of NB patient samples reveals that *FGFR2* correlates with *MYCN* amplification and advanced stage of NB [106]. No mutations of *FGFR3* have been found in NB patients, although publicly available NB sample data analysis shows that high *FGFR3* expression is associated with a worse event-free and overall survival [109]. It is only known that FGFR4 Arg388 polymorphism is associated with high occurrence of NB [110]. *FGF9* gene, which encodes a ligand for multiple FGFRs, including FGFR1 and FGFR2, is expressed by stromal cells in NB tumours [78]. FGF9 is known to promote the proliferation of neural progenitor cells [111], but its functional impact on NB cells remains unknown.

### 4.5. IGF-IR

Both IGF-I and IGF-II factors stimulate the growth of NB cell lines through the activation of signal cascades, including the phosphoinositide 3-kinase (PI3K)-protein kinase B (AKT) and mitogen-activated protein kinase (MAPK)-extracellular signal-regulated kinase (ERK) pathways [112]. Although higher expression of IGF-I/II receptor (IGF-IR) correlated with better prognosis for NB patients [11], IGF-IR plays a significant role in the proliferation of *ALK*-mutated NB cell lines via activation of PI3K-AKT and MAPK-ERK pathways [113]. IGF-I is secreted by TME cells: mesenchymal stromal cells [114], endothelial cells [115], eosinophils [116], and bone matrix (in case of osteolysis) [117]. It also promotes the activation of preosteoclasts [118] and high expression of *IGF1R* in NB cell lines is linked with bone metastasis [119].

In addition, ectopic expression of the *MYCN* gene leads to an increase in *IGF1R* expression [120]. NB cells’ stimulation with IGF-I leads to an increase in the expression of *MYCN* itself through activation of the MAPK signalling cascade [121]. Inhibition of IGF-IR leads to a slowdown in the growth of NB cells, including through phosphorylation and deactivation of N-MYC, is mediated by glycogen synthase kinase-3 β (GSK3β) [122]. It is possible that the simultaneous expression of *IGF1R* and *MYCN* leads to a mutual increase in the activity of these two proto-oncogenes, the formation of a positive feedback between them and contributes to the formation of aggressive forms of NB. Activation of TRKA by NGF leads to an increase in *IGF2* expression, and blocking of the IGF-IR by antibodies inhibits NGF-induced NB cell proliferation but does not affect NGF-dependent differentiation [123]. Thus, stimulating IGF-II synthesis may be one of the mechanisms by which NGF can promote the proliferation of neuroblast cells.

### 4.6. PDFR

Platelet-derived growth factor (PDGF) is traditionally considered to be a factor involved in tissue healing, stimulating cell proliferation, chemotaxis, and extracellular matrix production [124]. PDGF receptors (PDGFR) are expressed in NB cell lines and tumour tissues, and PDGFs themselves stimulate the growth and migration of these cells [125,126]. One isoform of PDGF, PDGF-A, is more highly expressed in NBs at stages 3 and 4 compared to stages 1, 2, and 4 s [127]. PDGF-BB is known to be secreted by endothelial cells [128] and preosteoclasts [129]. The addition of PDGF-BB to SH-SY5Y cells results in an increase in AKT and ERK protein phosphorylation. However, the role of PDGF in NB formation is not well understood. Although PDGF promotes the growth and migration of neuroblast cells and PDGFR may be a potential target for therapy, high expression of *PDGFRB* and *PDGFA* is correlated with increased patient survival [127,130].

### 4.7. EPOR

EPO is a glycoprotein hormone that is involved in erythropoiesis [131]. However, *EPOR* expression is not limited by haematopoietic cells and it is also expressed in neural cells [132]. Moreover, the neural crest is the first site of EPO production in mice embryos [133], and NCC derived from human induced pluripotent stem cells can secrete a substantial amount of EPO [134]. NB tumours can co-express EPO and its receptor, and their expression correlates with tumour angiogenesis [135], and EPO has been shown to induce mobility and adhesion in NB cell lines [136]. *EPO* expression is induced in SH-SY5Y and Kelly cells in response to hypoxia and EPO interacts with plasma membrane T-type voltage-dependent Ca^2+^ channels in NB cells [137,138]. Higher *EPOR* expression is associated with unfavourable prognosis both for MYCN non-amplified NB tumours and in relapsed tumours [139]. EPO displays cytoprotective properties in neural-like cells, as it prevents apoptosis induced by protein kinase C inhibitor staurosporine in differentiated SH-SY5Y cells [140]. Although EPOR activation does not promote NB cell proliferation or oncogenic signalling, it protects NB cells against etoposide and vincristine [141], and against multiple RTKi in an AKT- and ERK-dependent manner [139].

### 4.8. KIT

Immunohistochemical studies have found that 15–30% of all NBs are KIT-positive, while PCR data suggest that KIT is expressed in 40–80% of these tumours, depending on the specific study [142,143,144], and about half of KIT-positive NB tumours also express SCF [142]. There are conflicting data regarding the prognostic role of KIT. Some studies have shown that *KIT* expression correlates with a favourable prognosis and that KIT, as well as other genes such as *PDGFR*, are expressed in more differentiated NBs and in early-stage tumours [143]. Nevertheless, most studies suggest that *KIT* expression is associated with an unfavourable prognosis [20,139,142,144]. Among NB cell lines, a relatively low percentage of cells express KIT (5–20%), but *KIT* expression increases significantly under hypoxia conditions, and KIT is primarily expressed on surface of cancer stem cells [144]. Depletion of KIT-positive cancer stem cells significantly reduces tumour growth in mice; however, it is important to note that the presence of KIT alone is not sufficient for tumour formation [144]. KIT-positive neural crest cells give rise to melanocyte and cardiomyocyte lineages, which branch off during early neural crest development. This finding further supports the hypothesis that KIT-positive NB cells have stem-like properties and indicates that in these tumours, the oncogenic event may have occurred during early neural crest development. *KIT* knockdown in NB cell lines results in strong induction of apoptosis and induced mitotic catastrophe. Sensitivity to *KIT* depletion by shRNA in NB cells correlated with sensitivity to KIT-targeting multikinase inhibitors, such as sorafenib, imatinib, and pazopanib, suggesting that KIT is one of the primary targets of those inhibitors [20].

### 4.9. RET

RET is expressed in almost all NB tumours and cell lines [145,146]. However, no mutations of this gene are found in NBs [147]. *RET* expression is increased in NB cell lines by RA, and, furthermore, upregulated RET can induce differentiation in NB cells [148]. Also *RET* knockdown induces transition of NB cells to a mesenchymal phenotype, which generally is considered as more aggressive and resistant to treatment [149]. Conversely, some studies report that RET may take part in NB proliferation and metastasis [150,151]. A more detailed review of RET contribution to NB pathogenesis is provided in Rozen’s paper [11]. RET ligand, neurturin (encoded by the *NRTN* gene), is expressed by adrenocortical-like stromal cells in NB tumours, and its high expression correlates with a poor prognosis. Neurturin stimulates the growth and migration of NB cell lines, an effect that can be blocked by the RET inhibitor selpercatinib [152].

### 4.10. MET

Increased levels of mesenchymal–epithelial transition factor (*MET*) expression and amplification of this gene are found in advanced stage NB tumours [153,154]. NB cells can both secrete HGF and express MET, which indicates an autocrine loop, and hepatocyte growth factor (HGF) stimulated NB tumour angiogenesis in chick embryos [69]. Moreover, MET controls tumour and 3D spheroid tumour growth and its expression is associated with poor prognosis, tumour progression, relapse, and elevated levels of *MYCN* in patients [155].

### 4.11. AXL

AXL, a member of the TAM kinase family and a receptor for growth arrest-specific protein 6 (GAS6), is known to contribute to invasion, inhibition of apoptosis, increased proliferation, and chemoresistance in various cancers [156]. *AXL* expression in NB cells and tumours with a mesenchymal signature was higher than in tumours with adrenergic signature; however, AXL expression does not drive adrenergic-to-mesenchymal transition in NB cells [99]. AXL levels in mesenchymal-like SK-N-AS NB cells can be regulated by long non-coding RNA metastasis-associated lung adenocarcinoma transcript 1 (MALAT-1) and contribute to increased cell migration [157]. Single-cell data showed *AXL* expression in a tumour microenvironment [99], and given that AXL is capable of homophilic interaction if expressed on neighbouring cells, its expression could be responsible for paracrine interaction between tumour cells and cancer-associated microenvironments [158]. Moreover, AXL can be activated independently from GAS6 by forming homodimers, homophilic interactions, or heterodimers with other growth factor receptors, such as EGFR, HER2, MET, and PDGFR [158]. In heterodimer form, AXL phosphorylation can be triggered by activation of its partner receptor; for example, HGF induces phosphorylation of both MET and AXL when they form heterodimer [159]. AXL can also alter other receptor signalling; for example, EGF can activate pro-invasive genes via activation of EGFR-AXL heterodimer in glioblastoma cells [160]. Thus, AXL can potentially enhance the action of ERBB, MET, and PDGFRs in NB cells, since all these receptors are frequently overexpressed in NB cells. The AXL ligand GAS6 is secreted by cancer-associated fibroblasts in NSCLC [161] and by tumour-associated macrophages in bone marrow [162]; however, its secretion within NB tumours has not yet been established.

### 4.12. VEGFR

Vascular endothelial growth factor A (VEGF-A, also known as VEGF) is one of the most important factors for angiogenesis [163]. VEGFRs are differentially expressed in NB cell lines and patient samples [164,165,166]. As a regulator of angiogenesis, VEGF is usually associated with tumour progression, metastasis and drug resistance in many types of cancer [167,168]. However, the data for NB are controversial: it has been shown that VEGF-driven angiogenesis is involved in NB formation and metastasis [169,170]; however, the correlation between *VEGFA* expression and NB differentiation was identified [171]. It is stated that cell lines stimulate mesenchymal stromal cells to produce VEGF, which leads to promotion of osteogenesis [172]. Also, *MYCN*-amplified NB cell lines are known not only to produce VEGF, but also to stimulate VEGF production in TME cells [82]. It is considered that NB cells and hypoxia-inducible factor-2α (HIF-2α)-producing tumour-associated macrophages cooperate to promote angiogenesis, as they were found in perivascular niche, which is characterised by high levels of VEGF [116]. Cancer-associated fibroblasts secrete VEGF to promote metastasis by angiogenesis stimulation [173]. Single-cell analysis of myeloid population in metastatic NB samples shows that neutrophils have a high expression of *VEGFA* [174]. Inhibition of interaction between VEGFR3 and focal adhesion kinase (FAK) decreased NB cell survival [175], and also VEGF-C is considered to be a risk factor for stage IV NB [176].

A summary of the reviewed RTK and RTK genes expression in *MYCN*-amplified NB cells is provided in Table 1. Expression status in *MYCN*-amplified NB tumours was obtained from Cangelosi dataset [177] using R2 Genomics Analysis and Visualization Platform (https://hgserver1.amc.nl/ (accessed on 10 October 2025)); prognosis data were extracted from [11], or if no data were available, association with overall survival was calculated using R2 platform.

## 5. Inhibition of Growth Factor Signalling for Neuroblastoma Treatment

**Figure 2:**
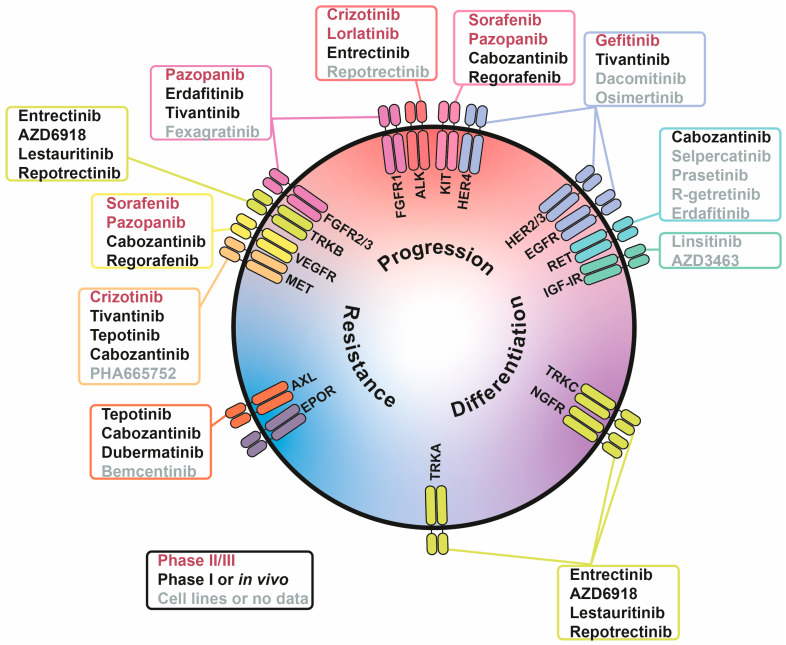
Involvement of receptor tyrosine kinases (RTKs) in tumour-associated processes in neuroblastoma (NB) cells. RTKs are positioned based on their involvement in three processes: NB progression (red), differentiation (purple), and drug resistance (blue). Inhibitors are written in three colours, indicating their stage of NB research: phase II/III (red), phase I or in vivo studies (black), and cell lines or no data (grey).

The majority of growth factor receptors are RTKs, which naturally makes RTKi a primary approach to target growth factor signalling in cancer. There are numerous RTKi in development; however, for safety reasons, drugs which have not been previously tested in humans are not generally approved for experimental treatments of childhood cancers, such as NB. Thus, in this review we give priority to drugs suitable for their repurposing to treat NB and focus on drugs which are already used for treatment of other cancers and have completed safety studies in NB patients (Table 2, Figure 2). Several inhibitors with limited data in NB models were also included, specifically those targeting RTKs with recently discovered roles in NB pathology or neural crest development. Although these RTK inhibitors are far from being adapted into NB treatment protocols, they are extensively studied in other cancer types and may represent a promising direction for future translational research (Table 3, Figure 2).

### 5.1. ALK Inhibitors

The most studied RTKi for NB treatment are ALK inhibitors, which are used for experimental treatment of ALK-mutant NB tumours. There are currently three generations of ALK inhibitors approved for treatment of NSCLC and anaplastic large cell lymphoma (ALCL): crizotinib (first generation), ceritinib, alectinib, brigatinib (second generation), and lorlatinib (third generation) [179]. Lorlatinib has shown a greater efficacy over crizotinib in the treatment of patients with advanced ALK-positive NSCLC [182]. ALK inhibitors are actively tested for NB treatment [75,222] but in most cases fail to completely eliminate tumours as a monotherapy, although lorlatinib showed promising results in combination with MIBG therapy [181]. Unlike NSCLC or ALCL, in which aberrant ALK activity is a result of chromosome translocation involving *ALK* gene, NB tumours have point mutations in the *ALK* gene or *ALK* overexpression, which might explain lower efficacy of ALK inhibitors in NB compared to other cancers.

### 5.2. TRK Inhibitors

Entrectinib, a ROS1, pan-TRK, and ALK inhibitor approved for treatment of tumours with *ROS1* or *NTRK* fusions [199] showed promising results in one NB patient with *ALK* mutation [223] (clinical trials Identifier: NCT02650401). AZD6918 is a potent TRKB inhibitor, which has failed phase I study for the treatment of adult patients with refractory solid malignancies (NCT00733031). However, AZD6918 has shown effectiveness in combination treatment with topoisomerase II inhibitor etoposide in TP3 mice xenografts [200]. Lestaurtinib, a multi-kinase inhibitor of TRK, FLT3, and JAK2 kinases, is a staurosporine derivative [224], and it was tested in phase III clinical trial for acute myeloid leukaemia (AML) with FLT3-ITD mutation treatment, although no significant improvement has been shown [184]. Moreover, lestaurtinib has been tested in phase I study in the treatment of recurrent and refractory high-risk NB and has shown to be well tolerated in patients [183]. Another pan-TRK inhibitor, repotrectinib, is approved for the treatment of adult patients with locally advanced or metastatic ROS1-positive NSCLC [202] and has shown effective inhibition of CLB-BAR tumour growth in mice, compared to crizotinib [201].

### 5.3. EGFR Inhibitors

EGFR and HER2 inhibitor, afatinib, blocks the growth of neuroblast cells in vitro and in vivo and restores NB cell sensitivity to doxorubicin [89]. It has been shown that simultaneous inhibition of several receptors from the ERBB family leads to a more significant effect on NB growth in vivo [90]. There are three generations of EGFR inhibitors. First (erlotinib and gefitinib) and second (afatinib) generations of drugs have been tested in clinical trials for NB treatment [185,225,226], but they failed to reach study endpoints. Dacomitinib (second generation), osimertinib, and nazartinib (third generation) have shown successful results for the treatment of EGFR-mutated NSCLC in phase II or III trials [203,204,227], although there are no data on these drugs being tested on NB cells.

### 5.4. FGFR Inhibitors

Fexagratinib (AZD4547) showed effectiveness in combination with PI3K inhibitor, dactolicib (BEZ235), in five NB cell lines (SK-N-AS, SK-N-BE(2)-C, SK-N-DZ, SK-N-FI, and SK-N-SH) [109]. However, phase II study of AZD4547 for the treatment of tumours harbouring aberrations in the FGFR pathway failed to reach its endpoints [228], and in phase I/II study, with FGFR fusion-positive glioma patients, fexagratinib has been beneficial, although additional investigation is required [205]. Another inhibitor, erdafitinib (JNJ-42756493), which is approved for the treatment of locally advanced or metastatic urothelial carcinoma [https://www.fda.gov/drugs/resources-information-approved-drugs/fda-approves-erdafitinib-locally-advanced-or-metastatic-urothelial-carcinoma (accessed on 10 October 2025)], has been tested on 2D and 3D NB models in combination with PI3K and cyclin-dependent kinase 4/6 (CDK4/6) inhibitors, alpelicib (BYL719) and palbociclib (PD-0332991), respectively. Combinations with erdafitinib have not been so effective [229], but erdafitinib and lorlatinib acted synergistically in decreasing tumour growth in patient-derived xenografts (PDX) models of high-risk *MYCN*-amplified and *ALK*^F1174L^-mutant NB [107].

### 5.5. IGF-IR Inhibitors

Although there are no IGF-IR inhibitors that have passed phase III studies, they still are considered as promising agents for combination therapy [230]. The combination of lorlatinib and IGF-IR inhibitors (GSK1904529A and linsitinib) decreases cell growth and MYCN protein level in ALK-driven *MYCN*-amplified NB cell lines [113]. MEK inhibitor, trametinib, combined with ganitumab, IGF-IR monoclonal antibody, suppressed proliferation and induced apoptosis in NB cell cultures but failed to reduce growth of primary or metastatic tumours in mice xenografts [231]. Also, there are data on another anti-IGF-IR monoclonal antibody effect: monoclonal antibody R1507 increases sensitivity to cisplatin in SH-SY5Y cells [232]. AZD3463, a dual ALK/IGF-IR inhibitor, suppresses NGP and SH-SY5Y tumours’ growth in mice xenograft [207].

### 5.6. RET Inhibitors

There are two highly selective RET inhibitors—selpercatinib and pralsetinib—which have shown efficacy against *RET*-mutated cancers [210,211]. R-getretinib, a novel RET inhibitor, has shown efficacy against NB cell lines, but the drug has not been tested against any other cancer or in mice xenografts yet [212].

### 5.7. MET Inhibitors

Tivantinib is a specific c-MET inhibitor, which has been tested in phase III clinical trial for the treatment of hepatocellular carcinoma, although no significant efficacy has been shown [188]. It inhibits cell growth and 3D spheroid tumour formation in different NB cell lines. Tivantinib induces apoptosis and blocks cell cycle progression at the G2/M phase transition in SH-SY5Y and NGP cells [155]. The drug has been tested in phase I trial; however, there were only two neuroblastoma patients, and neither showed significant response [187]. Another MET inhibitor—tepotinib (or EMD1214063)—approved for the treatment of NSCLC with MET alterations [214] inhibited MEK phosphorylation in SK-N-AS, SK-N-SH, and SH-EP cell lines and reduced mice xenograft tumour growth of SK-N-AS and SH-SY5Y cells [213]. There is also another inhibitor, PHA665752, which blocked HGF-induced phosphorylation of ERK and AKT in SH-EP cells [153]; however, its potential for in vivo use is controversial [233].

### 5.8. AXL Inhibitors

Bemcentinib, or R428, is a selective small molecule inhibitor of AXL, which effectively inhibits AXL phosphorylation in SK-N-AS and SH-EP-2 cells, and decreases the activity of AXL-dependent signalling pathways, such as PI3K- and ERK-pathways [157]. Bemcentinib has been tested in phase Ib/IIb trial in AML patients as monotherapy and in combination with low-dose cytarabine and has shown promising results [234]. Inhibition of AXL and related receptor tyrosine kinase MER in NB cells decreases proliferation, induces apoptosis, and increases sensitivity to cisplatin and vincristine [235]. There is also an AXL-targeted antibody-drug conjugate—ADCT-601 (mipasetamab uzoptirine). ADCT-601 is tested on several NB lines and decreases tumour volume in SK-N-AS mice xenografts [234]. Another AXL inhibitor, dubermatinib (or TP-0903), has passed phase I clinical trial for the treatment of patients with advanced solid tumours [219]. It was tested on SH-SY5Y cells and CDX mice models (Neura-2a xenografts), although AXL receptor is not present on the surface of these cells (https://depmap.org/portal/ (accessed on 10 October 2025)), which allows a conclusion that the drug inhibits other RTK in those NB cells [218].

### 5.9. Multikinase Inhibitors

Due to the high homology of RTK domains, many RTKi can target multiple receptors, which can be beneficial for simultaneous blocking of several overactivated receptors. For example, cabozantinib, which is approved by FDA to treat thyroid cancer, renal cell cancer (RCC), hepatocellular carcinoma (HCC), and neuroendocrine tumours [14], can target VEGFR2, MET, RET, KIT, and AXL. Cabozantinib inhibited RET signalling in NB cells [236], reduced tumour size and weight in SK-N-SH mice xenograft [236], inhibited tumour growth in genetically engineered mouse models of NB [237], demonstrated clinical benefit in four patients with relapsed high-risk NB [189], and currently undergoes phase II study as a maintenance agent for NB (NCT05135975). Sunitinib, a PDGFR, VEGFR, and KIT inhibitor, approved for RCC, gastrointestinal stromal tumour (GIST), and pancreatic neuroendocrine tumour [14], suppresses growth of *MYCN*-amplified NB tumours in mice xenograft [220]. Sorafenib, a B-rapidly accelerated fibrosarcoma (B-RAF), C-RAF, KIT, PDGFR, VEGFR, and FMS-like tyrosine kinase 3 (FLT3) inhibitor approved for the treatment of RCC [191] and advanced HCC [192], inhibited NB tumour growth in CDX models (SMS-KCNR xenografts) [238]. However, NB patients treated with sorafenib had progressive disease, despite the good tolerability of sorafenib [190]. Regorafenib, a sorafenib derivative, approved for the treatment of HCC [https://www.fda.gov/drugs/resources-information-approved-drugs/regorafenib (accessed on 10 October 2025)], GIST [195], and metastatic colorectal cancer [196], inhibits RET and PDGFRβ in NB cells, leading to decreased proliferation and colony formation ability and reduced tumour growth in CDX models [239,240]. Phase I study of regorafenib has shown its efficacy in the treatment of paediatric patients with relapsed/refractory solid tumours, alone and in combination with backbone therapy (vincristine and irinotecan), but has not been effective for one NB patient included in this study [193,194]. Axitinib, a VEGFR, PDGFR, and KIT inhibitor approved to treat patients with RCC [14], inhibits NB tumour growth in vivo [221]. Pazopanib, a VEGFR, PDGFR, FGFR1-2, and KIT inhibitor, is approved for the treatment of soft-tissue sarcoma [197] and advanced RCC [198]. It delayed tumour growth in CDX models (SK-N-BE(2) xenografts) in combination with topotecan and showed some antiangiogenic activity, but tumours tend to develop resistance, perhaps due to elevated glycolysis [241]. Phase II clinical trial to identify the efficacy of pazopanib alone in solid paediatric tumours, including four NB patients, has been performed, but it failed to prevent disease progression (NCT01956669). Many multikinase inhibitors, such as cabozantinib, sorafenib, axitinib, and pazopanib, target one or several receptors from VEGFR family, and thus inhibit tumour angiogenesis in addition to killing cancer cells. Also, those inhibitors have a diverse impact on immune cells, which can promote or inhibit anticancer immune response [242].

**Figure 3:**
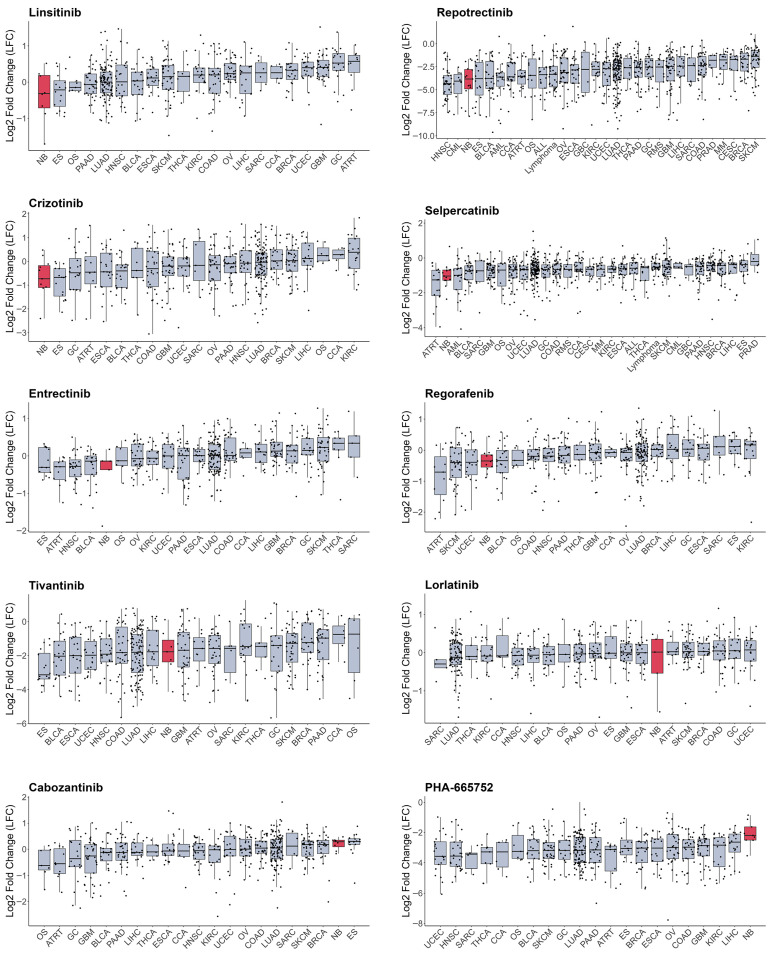
Cell number change in log2 after treatment with RTK inhibitors from PRISM database. Cell lines grouped by primary tumour type, neuroblastoma cells marked in red. Median values and lower and upper quartile are shown; each dot represents a cell line. CCA—cholangiocarcinoma, BLCA—bladder urothelial carcinoma, BRCA—breast invasive carcinoma, OS—osteosarcoma, CESC—cervical squamous cell carcinoma and endocervical adenocarcinoma, COAD—colon adenocarcinoma, UCEC—uterine corpus endometrial carcinoma, ESCA—esophageal carcinoma, ES—Ewing sarcoma, GBC—gallbladder cancer, GC—stomach adenocarcinoma, GBM—glioblastoma multiforme, HNSC—head and neck squamous cell carcinoma, KIRC—kidney renal clear cell carcinoma, LIHC—liver hepatocellular carcinoma, LUAD—lung adenocarcinoma, SKCM—skin cutaneous melanoma, MM—multiple myeloma, NB—neuroblastoma, OV—ovarian serous cystadenocarcinoma, PAAD—pancreatic adenocarcinoma, PRAD—prostate adenocarcinoma, ATRT—atypical teratoid rhabdoid tumour, RMS—rhabdomyosarcoma, SARC—sarcoma, THCA—thyroid carcinoma, CML—chronic myeloid leukaemia, ALL—acute lymphoblast leukaemia, AML—acute myeloid leukaemia.

**Figure 4:**
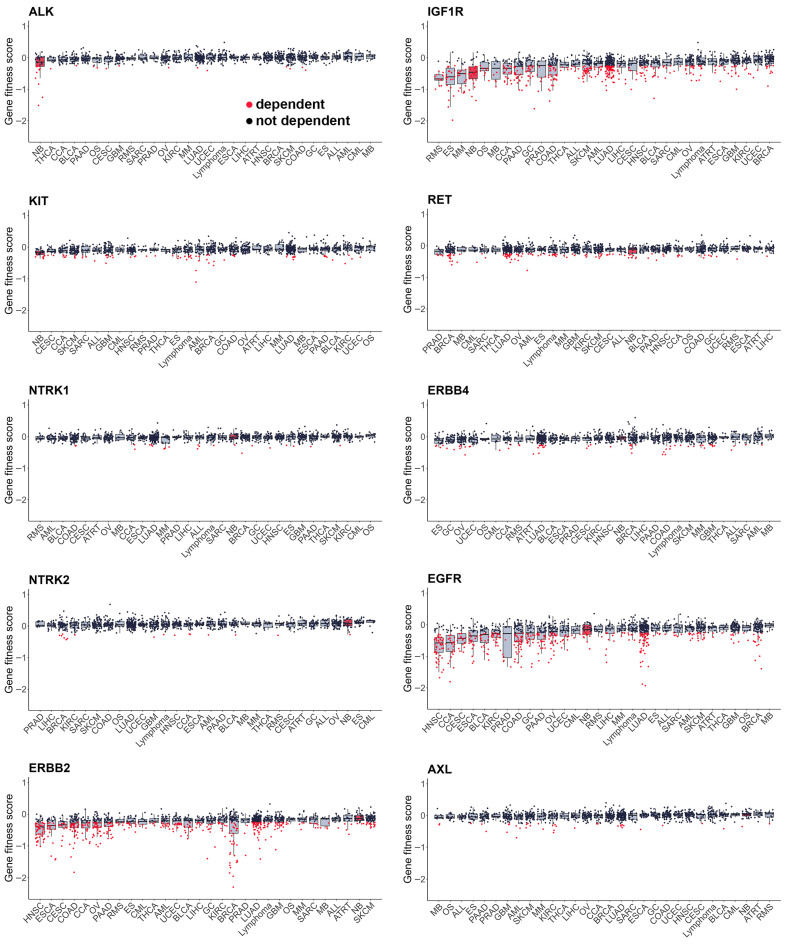
Gene fitness scores combined from CRISPR and RNAi screens from DepMap database. Cell lines grouped by primary tumour type, neuroblastoma cells marked in red. Median values and lower and upper quartile are shown; each dot represents a cell line. CCA—cholangiocarcinoma, BLCA—bladder urothelial carcinoma, BRCA—breast invasive carcinoma, OS—osteosarcoma, CESC—cervical squamous cell carcinoma and endocervical adenocarcinoma, COAD—colon adenocarcinoma, UCEC—uterine corpus endometrial carcinoma, ESCA—esophageal carcinoma, ES—Ewing sarcoma, GBC—gallbladder cancer, GC—stomach adenocarcinoma, GBM—glioblastoma multiforme, HNSC—head and neck squamous cell carcinoma, KIRC—kidney renal clear cell carcinoma, LIHC—liver hepatocellular carcinoma, LUAD—lung adenocarcinoma, MB—medulloblastoma, SKCM—skin cutaneous melanoma, MM—multiple myeloma, NB—neuroblastoma, OV—ovarian serous cystadenocarcinoma, PAAD—pancreatic adenocarcinoma, PRAD—prostate adenocarcinoma, ATRT—atypical teratoid rhabdoid tumour, RMS—rhabdomyosarcoma, SARC—sarcoma, SCC—squamous cell carcinoma, THCA—thyroid carcinoma, CML—chronic myeloid leukaemia, ALL—acute lymphoblast leukaemia, AML—acute myeloid leukaemia.

As most RTKi target different sets of kinases, it is hard to estimate and compare their selectivity towards NB cells. To compare selectivity of reviewed compounds we utilised PRISM drug repurposing dataset, which compares drug efficacy at single dose across 859 cancer cell line of different origin (downloaded from DepMap portal https://depmap.org/portal/ (accessed on 10 October 2025)) [243]. Interestingly, crizotinib, a first-generation ALK inhibitor, showed high selectivity towards NB cell lines, while third-generation ALK lorlatinib does not have such selectivity (Figure 3, Table S1). Although lorlatinib is a more selective inhibitor of mutant ALK, crizotinib might display more activity towards NB cells, since most NB cells have ALK overactivation rather than *ALK* mutations. Crizotinib also inhibits MET [244], which can contribute to its activity against NB cells, where ALK and MET can be simultaneously active. The second drug, which showed the highest selectivity, was IGF-1R inhibitor linsitinib; however, dual ALK/IGF-1R inhibitor AZD3463 showed low selectivity towards NB cells. Among other drugs with high selectivity were drugs that target RET, such as repotrectenib and selpercatinib. Drugs which primarily target MET, such as PHA665752, tivantinib, and cabozantinib, or FGFR inhibitors erdafitinib and pazopanib, showed low selectivity. Surprisingly, although cabozantinib has been shown to inhibit RET phosphorylation in NB cells [236], and showed benefits for maintenance of high-risk NB patients, it did not show selectivity towards NB cell lines. To compare the importance of individual RTKs for NB survival we used DepMap data on gene depletion effects on cell proliferation [21] by combining data for CRISPR and RNAi screens as described in our paper [20] (Figure 4). *ALK*, *IGF1R*, and *KIT*, but not *MET* or *RET*, depletion caused reduced NB cell proliferation and had selective effect for NB cells. These data suggest that drugs which selectively target ALK, IGF-1R, or KIT can potentially have more profound effects on NB cell lines. Interestingly, although RET inhibitors repotrectenib and selpercatinib are selective for NB cell lines, *RET* gene depletion did not have selective effect on NB cells, suggesting that those inhibitors may act through additional targets. Additional inhibition of kinases implicated in drug resistance or ability of cancer cells to adapt, such as TRKs by entrectinib or repotrectinib, MET by crizotinib, and MAPK pathway by regorafenib or sorafenib, may also enhance drug action against NB.

Still, large-scale drug screens on cell lines, such as PRISM, have several important limitations. Cell lines often have different frequency of oncogenic mutations compared to real tumours. NB cell lines have much higher frequency of TP53 and ALK mutations, while ATRX alterations are underrepresented [139], which may introduce additional bias into evaluation of drug selectivity towards NB cells. Also, only some NB cell lines have a MES phenotype, which restricts statistical power for search of drugs that can eliminate both MES and ADRN cells. This may also explain low median gene fitness scores of MES-specific genes, such as *ERBB3/4* and *AXL*. PRISM dataset was selected for drug sensitivity analysis, because it provides the best coverage of RTK inhibitors, with 1514 compounds screened on 859 cell lines, but cells were treated with a single drug dose, which limits the ability to determine overall cell line sensitivity to a certain drug.

## 6. Targeting Resistance to RTK Inhibitors in Neuroblastoma

The major obstacle in the use of RTKi is the development of drug resistance. As described in this review, RTKi, despite promising results in cell and xenograft models, usually show short-term effectiveness in patients, and most fail to induce complete response. Still, RTKi remain promising agents for NB therapy, especially if used in combination with drugs that target resistance development. The origins of resistance to RTKi in neuroblastoma are poorly understood, and data on resistance in patients are available only for patients treated with ALK inhibitors. Also, only patients with *ALK* mutations are treated with ALK inhibitors in clinical trials, and *ALK* mutations occur in about 9% of NB; however, ALK or other RTK overactivation occurs almost in the majority of NB cases. Mutations in neurofibromatosis-related protein (*NF1)*, *NRAS,* and *HRAS* genes were found in tumours that developed resistance to lorlatinib or ceritinib, and NF1 loss conferred resistance to ALK inhibitors in NB cells [245]. However, this study was limited to only four patients and, in general, mutations in *NF1* or *NRAS* in NB tumours occur rarely [147,246]. Thus, we discuss possible non-genetic mechanisms of acquired or pre-existing resistance to RTKi, and due to the scarcity of NB patient data, we rely mostly on in vitro and in vivo studies, or data from other cancers where the use of RTKi is more widespread.

### 6.1. Bypass Signalling by Other Growth Factor Receptors

**Figure 5:**
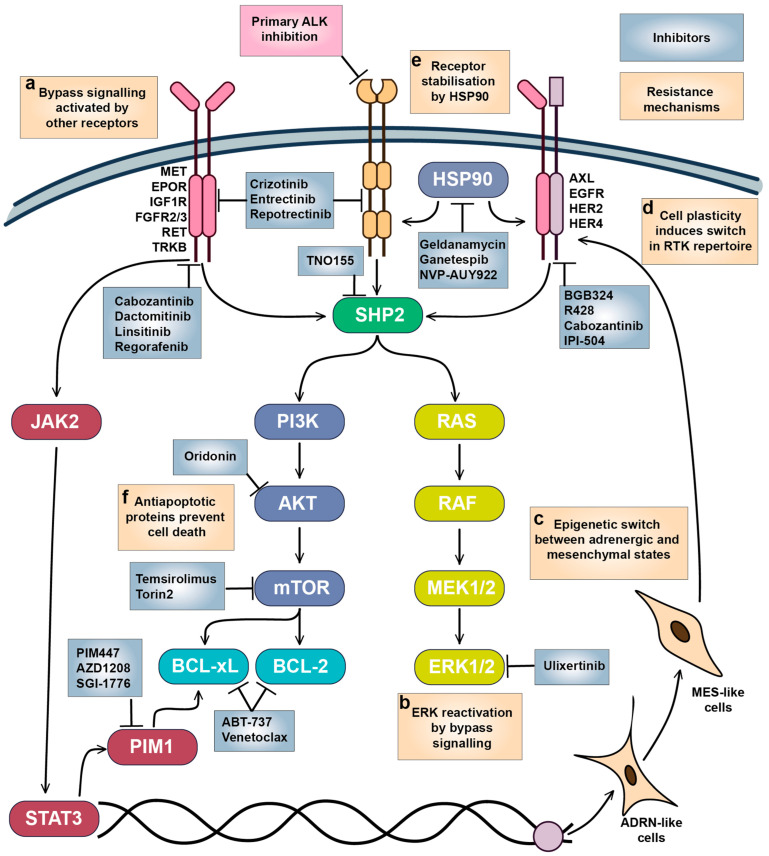
Mechanisms of adaptive resistance to RTKi in neuroblastoma. (**a**) Bypass signalling activated by other growth factors and their receptors reactivated downstream pathways required for NB survival. (**b**) ERK1/2 reactivation promotes cell survival and proliferation, which is likely triggered by bypass signalling through alternative RTKs. (**c**) Epigenetic switch and (**d**) cell plasticity between ADRN and MES-like states triggers expression of a different set of RTKs. (**e**) RTK stability and maturation are controlled by HSP90, which also modulates bypass signalling. (**f**) Overactivation of antiapoptotic signalling maintains NB cell survival. Inhibitors targeting the main vulnerabilities in NB cells and tested on NB models are shown.

Activation of bypass signalling is the most described mechanism for RTK resistance [247,248] after acquirement of secondary mutations, which rarely happens in NB tumours. Usually, bypass to inhibition of RTK signalling is mediated by activity of other RTKs that activate the same or alternative downstream pathways and promote cell survival (Figure 5a). In vitro studies showed that growth factors such as EPOR, IGF-1, BDNF, NGF, and SCF can reduce efficacy of RTKi in NB cells and compensate for loss of KIT receptor signalling. MET activation has been shown to compensate for inhibition of mutant EGFR, or ALK, ROS1, and RET fusion oncogenes; MET inhibition is beneficial to overcome resistance in NSCLC [249,250]. Although there are no data on impact of MET inhibition on ALK resistance in NB, crizotinib, which is a dual ALK and MET inhibitor, shows high selectivity towards NB cells and was relatively successful in clinical trials. Developed resistance to ALK and pan-TRK inhibitor entrectinib in *ALK^F1174L^* NB xenografts was associated with increased TRKB or IGF1R activation [251], and resistance to lorlatinib in NB cells was accompanied by EGFR and HER4 overactivation [252]. EGFR or HER4 inhibition may also be an effective strategy to overcome ALK inhibitor resistance in NB, as EGFR activation by HB-EGF caused adaptive resistance to lorlatinib in NSCLC [253], and pan-HER inhibitor dactomitinib prevented the emergence of drug tolerant persister cells in ALK-positive NSCLC [254].

ERK1/2 reactivation is one of the most frequent events associated with resistance to RTKi (Figure 5b). Activation of ERK1/2 by RTKs rescues NB cells from a variety of RTKi [139,251]; MAPK/ERK pathway activity is lower in therapy-responsive PDX tumours [255], and RAS/MAPK signalling is increased in lorlatinib-resistant cells [256]. ERK1/2 inhibition by ulixertinib prevents the protective action of growth factors, shows synergy with RTKi in NB cells [139], and was able to reduce NB tumour growth in mice xenografts [257]. SHP2 phosphatase is a major regulator of MAPK activation by RTKs, and SHP2 inhibition by TNO155 enhances the action of ALK inhibitors in cell lines and zebrafish or murine xenografts [256]. ERK1/2 inhibitor ulixertinib showed an acceptable safety profile in patients with MAPK-mutant solid tumours [258], but there are still no data on ERK1/2 inhibitors tolerability in paediatric tumours.

### 6.2. Targeting Intratumoural Heterogeneity

Increasing evidence shows that AXL may act as master regulator of drug resistance to various RTKi through modulation of other RTK activities or promotion of EMT [158,248,259]. AXL overactivation was linked to resistance to ALK inhibitors in NB cells, which was also accompanied with EMT [260], and AXL inhibition or genetic depletion increased sensitivity to crizotinib [216]. Adrenergic (ADRN) to mesenchymal (MES) transition is thought to be a major driver in NB tumour heterogeneity [261,262], as cells with MES-like and ADRN signatures coexist within the same tumours and can transition between states [18,263]. Relapsed and high-risk NB tumours have higher presence of cells with MES signature, and MES cell lines are resistant to chemotherapy drugs [262]. MES and ADRN cells have distinct patterns of RTK expression: while ADRN cells express ALK, MES cells express high levels of *AXL*, *EGFR*, *PDFGRA*, *PDGFRB*, and *ERBB4* [18,99]. ADRN-MES transition in NB cell lines also leads to a loss of *ALK* expression, an increase in *AXL* expression, and loss of sensitivity of MES NB cells to ALK inhibition [99,264]. Although *AXL* expression was not essential for initiation of ADRN-MES transition [99], AXL can be a promising target for elimination of NB cells with MES signature resistant to ALK inhibitors (Figure 5c,d).

Many RTKs are client proteins of heat shock protein (HSP90) chaperone, which promotes their maturation, stability, and control protein export, and surface expression of HSP90 was found in NB cells, indicating its direct interaction with RTKs in NB [265,266] (Figure 5e). ALK-resistant SH-SY5Y NB cells were more sensitive to HSP90 inhibition by geldanamycin or IPI-504 than ALK-sensitive cells, and IPI-504 reduced AXL protein levels and ERK phosphorylation [260]. Geldanamycin destabilised TRKAI isoform localisation on NB cell surface and reduced TRKAIII splice variant phosphorylation [266], reduced protein levels of IGF1R and KIT in Ewing sarcoma cells, and improved IGF1R inhibitor activity in murine xenografts [267]. Another HSP90 inhibitor NVP-AUY922 reduced MET, HER2, HER4, EGFR, and AXL in ovarian cancer [268]. There are multiple HSP90 inhibitors in clinical development, extensively described in Rastogi’s paper [269], including ganetespib, which also induces ALK degradation, overcomes ALK resistance in NSCLC cells, and showed activity in NSCLC patient with prior crizotinib resistance [270]. HSP90 inhibitors geldanamycin and ganetespib as monotherapy reduced NB tumour growth in vivo, although overall response was moderate [271,272]. HSP90 inhibitors have been tested in numerous adult cancer trials, with new generation agents like ganetespib demonstrating reduced toxicity compared to their first-generation counterparts. Although paediatric tumours did not respond to the HSP90 inhibitor 17-AAG as a single agent, it was well-tolerated in children [273]. However, a lack of tolerability data for newer HSP90 inhibitors in children currently limits their potential use in NB treatment.

### 6.3. Antiapoptotic Signalling as Vulnerability in Neuroblastoma

**Figure 6:**
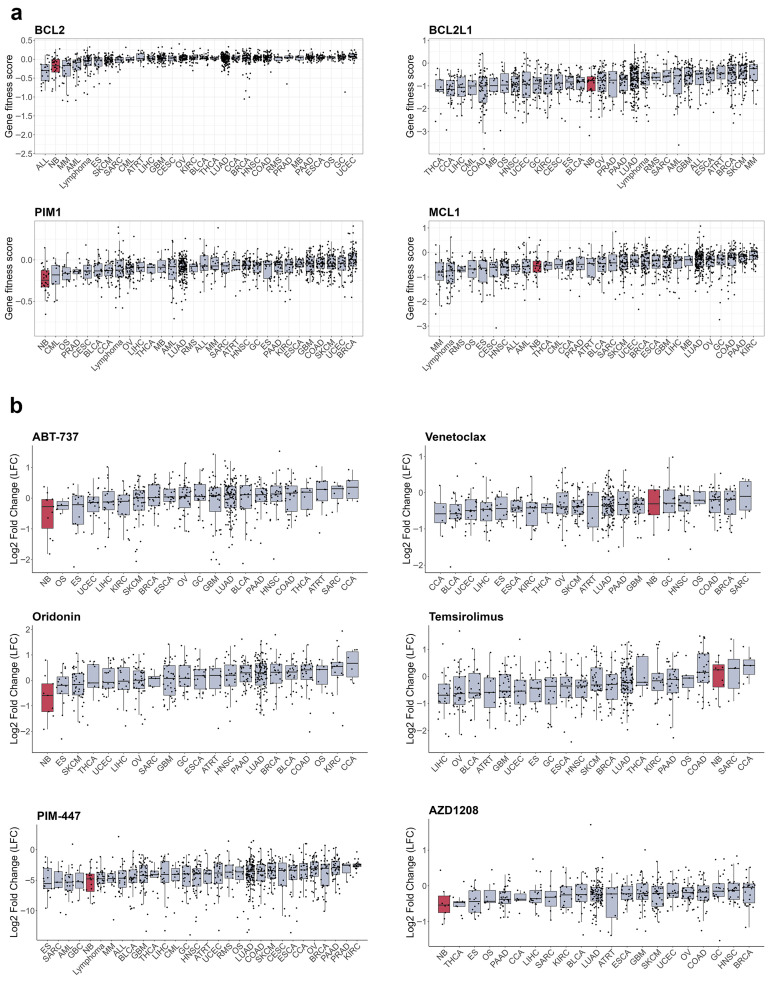
Sensitivity of cancer cell lines to apoptosis inducers. (**a**) Gene fitness scores combined from CRISPR and RNAi screens from DepMap database. (**b**) Cell number change in log2 after treatment with RTK inhibitors from PRISM database. Cell lines grouped by primary tumour type, neuroblastoma cells marked in red. Median values and lower and upper quartile are shown; each dot represents a cell line. CCA—cholangiocarcinoma, BLCA—bladder urothelial carcinoma, BRCA—breast invasive carcinoma, OS—osteosarcoma, CESC—cervical squamous cell carcinoma and endocervical adenocarcinoma, COAD—colon adenocarcinoma, UCEC—uterine corpus endometrial carcinoma, ESCA—esophageal carcinoma, ES—Ewing sarcoma, GBC—gallbladder cancer, GC—stomach adenocarcinoma, GBM—glioblastoma multiforme, HNSC—head and neck squamous cell carcinoma, KIRC—kidney renal clear cell carcinoma, LIHC—liver hepatocellular carcinoma, LUAD—lung adenocarcinoma, MB—medulloblastoma, SKCM—skin cutaneous melanoma, MM—multiple myeloma, NB—neuroblastoma, OV—ovarian serous cystadenocarcinoma, PAAD—pancreatic adenocarcinoma, PRAD—prostate adenocarcinoma, ATRT—atypical teratoid rhabdoid tumour, RMS—rhabdomyosarcoma, SARC—sarcoma, SCC—squamous cell carcinoma, THCA—thyroid carcinoma.

B-cell lymphoma 2 (BCL-2) homology 3 (BH-3) mimetics which target BCL-2, B-cell lymphoma-extra large (BCL-xL), and MCL-1 antiapoptotic proteins, are actively used in the treatment of hematologic malignancies and increased activity of those proteins has been linked to RTK resistance in various cancers [274,275,276] (Figure 5f). Various BCL-2, BCL-xL, and MCL-1 inhibitors induce apoptosis in NB cells [277,278,279], and BCL-2 mRNA and protein levels are increased in *ALK^F1174L^*/*MYCN* NB tumours compared to tumours with only *MYCN* amplification [280]. NB cells show high dependency on *BCL2* expression (Figure 6a), and remarkable sensitivity to the BCL inhibitor ABT-737 [279] and the AKT/BCL2 inhibitor oridonin [281] (Figure 6b). The impact of BH-3 mimetics on RTKi resistance in neuroblastoma remains unexplored, but BH-3 mimetics enhanced MET inhibition by crizotinib in glioblastoma PDX [282] and ALK inhibition by lorlatinib in NSCLC xenografts [275]. Notably, sensitivity of NB organoids to BCL2 inhibitor venetoclax correlated with mesenchymal expression signature, suggesting that BCL2 inhibition could also target intratumoural heterogeneity [152]. Venetoclax, extensively studied in childhood leukaemias, has a good safety profile [283]. Although not yet tested with ALK inhibitors, venetoclax has been used alongside kinase inhibitors, such as the FLT3 inhibitor midostaurin, for adult AML treatment [284]. These data suggest that BCL2 inhibitors, such as venetoclax, might be well tolerated in NB patients.

BCL-2 activity is directly controlled by AKT/mammalian target of rapamycin (mTOR) signalling, and a combination of crizotinib with mTOR inhibitor Torin2 prolonged survival of mice with ALKF1174L/MYCN NB tumours, which overexpress BCL-2 and are resistant to crizotinib [280]. A phase I/II study of crizotinib in combination with another mTOR inhibitor, temsirolimus, in relapsed or refractory neuroblastoma with ALK or MET aberrations is currently ongoing, although unexpected toxicity caused by temsirolimus was detected (EUCTR2015-005437-53). Still, mTOR inhibitors combined with chemotherapy show good tolerability in paediatric patients [285,286], and a ceritinib combination with everolimus is currently in clinical trials for adult NSCLC (NCT02321501). Another major regulator of cell survival is serine/threonine kinase PIM1, which can phosphorylate and shut down pro-apoptotic protein BCL-2 associated agonist of cell death (BAD), and thus also regulates BCL protein activity [287]. PIM1 kinase is constantly active, and its activity is controlled by protein stability or its expression, which is driven by Janus kinase 2 (JAK2)/signal transducer and activator of transcription 3 (STAT3) pathway. STAT3 involvement in cancer drug resistance is well known, including NB [288], JAK2/STAT3 signalling can be activated by a variety of RTKs, such as EGFR [289], MET [290], or AXL [158]. Although STAT3 inhibitors are only in early stages of clinical development and have not been tested on NB models, currently there are several bioavailable PIM1 inhibitors in clinical studies, including PIM447, AZD1208, and SGI-1776 [287]. NB cells show high sensitivity to PIM1 depletion (Figure 6a) and PIM inhibitors PIM447 and AZD1208 (Figure 6b), which showed activity in NB xenografts [291]. Genome-wide CRISPR activation screen in NB cells revealed that PIM1 drives resistance to ALK inhibitors brigatinib or ceritinib, and the combination of AZD1208 with ceritinib significantly delayed tumour growth in ALK mutant NB PDX models [292]. PIM1 inhibition was also beneficial to overcome resistance to MET or EGFR inhibitors in NSCLC [293,294], highlighting the potential of PIM1 inhibition in combination with various RTKi. Currently, PIM inhibitors have limited potential for NB treatment, as they have only recently entered clinical trials. The PIM1 inhibitor PIM447 was well-tolerated in patients with multiple myeloma [295]. However, more data on its safety profile are needed before clinical trials can be conducted for paediatric patients.

## 7. Conclusions

Current understanding of NB development indicates that malignant transformation can occur at least during several stages in neural crest development. Although RTK genetic alterations in NB are limited to 7–10% tumours with *ALK* mutations, the NB origin from NCC gives NB tumours a wide repertoire of targetable RTKs. Overview of published research, gene fitness and drug screen studies show that multiple RTKs, such as ALK, RET, and IGF1R, represent targetable vulnerabilities in NB cells. Current data show the potential of RTKi not only for ALK mutant NBs, but also for the majority of NB tumours with overactivation of growth factor signalling. Single-cell studies have expanded our understanding of neural crest development and revealed AXL and HER4 as some of the markers of transient cell states; however, the role of their activation or their ligands in neural crest development remains unclear. However, it remains largely unexplored which oncogenic signalling is controlled by AXL, HER4, or EGFR in MES-like NB cells, and what impact it has on survival of those cells and NB initiation in general. Repurposing RTKi for NB treatment is a compelling approach, as drug development is a time-consuming and costly process, whereas RTKi represent a vast pool of agents already tested in clinical trials. However, drug repurposing for NB has several limitations. Drug sensitivity data are mostly derived from screenings on NB cell lines, which possess limited genetic and epigenetic diversity, and most RTKis lack reliable paediatric toxicity data. The further development of clinically relevant models, such as drug screening on organoids [152] or PDX [255], could improve the effectiveness of this strategy and help overcome some of these limitations.

Intratumoural heterogeneity poses a significant challenge for the efficacy of both chemotherapy and targeted inhibitors [296]. In NB, this heterogeneity originates from a complex interplay of developmental cell states [297], epigenetic plasticity regulated by core regulatory circuitries [263,298,299], and clonal evolution. NB tumours are composed of multiple genetically distinct subclones, and evidence suggests such heterogeneity can be both pre-existing and therapy-induced [300]. Developmental states may contribute significantly to pre-existing heterogeneity, as malignant transformation can occur at different stages of neural crest development. For example, ALK-positive NB cells are thought to arise from sympathoblasts or chromaffin cells, HER3/4-positive cells from bridge cells, and KIT-positive cells from more stem-like early neural crest cells. As tumours undergo clonal evolution, the accumulation of genetic and epigenetic changes can impair differentiation. This process, along with the ADRN-to-MES transition, ultimately results in a tumour composed of multiple cell types, each with a unique RTK expression signature.

This heterogeneity has direct functional consequences for patient therapy. For example, ALK inhibitors primarily eradicate proliferating sympathoblast-like cells but are ineffective against MES-like cells. Resistant subclones, pre-existing or emerging during treatment due to high cellular plasticity, can subsequently drive refractory or relapsed disease. Furthermore, intratumoural heterogeneity can lead to inaccurate risk assessment and suboptimal treatment when diagnosis relies on a single-site biopsy. For instance, the spatial and temporal heterogeneity of actionable mutations in genes like *ALK* and *FGFR1* can compromise the accurate assessment of a tumour’s susceptibility to targeted inhibitors [300]. Collectively, these factors may explain why most RTKi have failed as monotherapies in NB clinical trials.

To translate RTKi effectively into NB treatment protocols, it seems necessary to assess the tumour heterogeneity of individual patients using techniques such as single-cell RNA sequencing, spatial transcriptomics, or proteomics. Such profiling could then guide combination therapies, based on RTKi that target receptors essential for the majority of ADRN-like cells and inhibitors of proteins upregulated in MES-like cells, such as AXL or BCL-2. A major concern for these novel combinations is their unknown toxicity, as none have reported clinical toxicity data in paediatric patients. Nevertheless, extensive safety data exist for adult patients treated with mTOR, BCL2, and HSP90 inhibitors, and some have also been tested in paediatric cohorts. The feasibility of drug repurposing for NB remains unclear due to a lack of direct clinical evidence, and most drug combinations require prior in vivo testing in NB models to demonstrate efficacy. However, based on the existing data, RTKi-based treatment represents a promising direction for NB translational research.

## Figures and Tables

**Table 1:** Characterisation of RTK involved in NB development. Table describes gene expression in *MYCN*-amplified NB cells, prognosis, role in NB cells, and connection with the resistance. FMS-related receptor tyrosine kinase 1 (*FLT1*) encodes VEGF1, kinase insert domain receptor (*KDR*) encodes VEGFR2, and FLT4 encodes VEGFR3.

Gene Name	Expression in *MYCN*-Amplified NB Cells	Prognosis	Role in NB Cells	Resistance to the Drugs
*ALK*	Higher	Unfavourable	One of the drivers of NB development and progression	Erdafitinib, GSK1904529A, linsitinib
*NTRK1*	Lower	Favourable	Plays a role in NB regression and differentiation	Resistance via PI3K/Akt, MAPK/ERK pathways
*NTRK2*	Higher	No association	Increases the survival of neuroblast cells and promote metastasis	Entrectinib
*NTRK3*	Lower	No association	Inhibition leads to the induction of apoptosis and inhibition of xenograft tumour growth	Resistance via PI3K/Akt, MAPK/ERK pathways
*NGFR*	Lower	Favourable	Reduces the rate of cell proliferation and increases the number of apoptotic cells in vitro, and prevents tumour formation in vivo	Resistance via PI3K/Akt, MAPK/ERK pathways
*EGFR*	Lower	No association	Stimulates NB cell proliferation in vitro, and EGFR inhibition by gefitinib induces apoptosis in NB cell lines	Lorlatinib, dactomitinib, osimertinib
*ERBB2*	Lower	No association	Highly expressed on NB cells with upregulated mesenchymal gene signature	Dactomitinib
*ERBB4*	Higher	No association	Highly expressed on NB cells with upregulated mesenchymal gene signature and detected in NB cells migrating along nerves in avian embryo	Lorlatinib, dactomitinib
*FGFR1*	NA	No association	High levels correlate with low relapse-free survival, silencing by shRNA inhibits cologenicity and invasion in SH-SY5Y and SK-N-BE2 cells, and overexpression of *FGFR1^N546K^* promotes cell invasion and colonigenicity	Resistance via PI3K/Akt, MAPK/ERK pathways
*FGFR2*	Higher	Unfavourable	Silencing leads to sensitisation to cisplatin of cisplatin-resistant NB cells	Lorlatinib, cisplatin
*FGFR3*	No data	No association	Expression is associated with a worse event-free and overall survival	Resistance via PI3K/Akt, MAPK/ERK pathways
*FGFR4*	Lower	No association	Arg388 polymorphism is connected with high occurrence of NB	Resistance via PI3K/Akt, MAPK/ERK pathways
*IGF1R*	Lower	Favourable	Plays a significant role in proliferation of *ALK*-mutated NB cell lines via activation of PI3K-AKT and MAPK-ERK pathways	Cisplatin, entrectinib, lorlatinib
*PDGFRA*	Higher	Unfavourable	Stimulates the growth and migration of NB cells	Resistance via PI3K/Akt, MAPK/ERK pathways
*PDGFRB*	Lower	No association	Stimulates the growth and migration of NB cells, high expression is correlated with increased patient survival	Resistance via PI3K/Akt, MAPK/ERK pathways
*EPOR*	Lower	Unfavourable	Expression of the receptor and its ligand correlates with tumour angiogenesis, EPO has been shown to induce mobility and adhesion in NB cell lines	Etoposide, vincristine
*KIT*	Lower	Unfavourable	Knockdown in NB cell lines results in strong induction of apoptosis and induced mitotic catastrophe	Sorafenib, imatinib, pazopanib
*RET*	Higher	Unfavourable	Upregulated RET can induce differentiation in NB cells, knockdown induces transition of NB cells to a mesenchymal phenotype, may take part in NB proliferation and metastasis	Resistance via PI3K/Akt, MAPK/ERK pathways
*MET*	NA	Unfavourable	HGF stimulated NB tumour angiogenesis in chick embryos, MET controls tumour and 3D spheroid tumour growth, its expression is associated with tumour progression, relapse and elevated level of *MYCN* in patients	Sotorasib, poziotinib, entrectinib
*AXL*	Lower	Favourable	Contributes to increased cell migration, inhibition of AXL in NB cells decreases proliferation, induces apoptosis, expressed in tumour microenvironment	ALK inhibitors, cisplatin, vincristine, crizotinib
*FLT1*	NA	No association	Involved in NB formation, metastasis and differentiation	Resistance via PI3K/Akt, MAPK/ERK pathways
*KDR*	Lower	Favourable		
*FLT4*	Lower	No association		

**Table 2:** RTK inhibitors and their application in neuroblastoma in completed trials and other types of cancer. The table describes drug targets, research progress in NB with relevant clinical trial numbers where applicable, and research progress in other cancers. RP2D—recommended phase II dose, ORR—objective response rate.

Drug	Target	Stage of Research in Neuroblastoma	Stage of Research in Other Types of Cancer
Crizotinib	ALK, ROS1, c-MET	Phase II Study (NCT00939770) ORR was 15% [178]	Approved by FDA to treat ALK-positive NSCLC and ALCL [179,180]
Lorlatinib	ALK, ROS1	Phase I Study Trial is completed, RP2D in children was 115 mg/m^2^, PR2D in adults was 150 mg; single-agent response rate for <18 years was 30%; for ≥18 years, 67%; and for chemotherapy combination in <18 years, 63% (NCT03107988) [181] Phase III Study Trial is active, patients have high-risk neuroblastoma (NCT03126916)	Approved in EU for NSCLC [179] Phase III CROWN Study Compared with crizotinib in patients with ALK-positive NSCLC (more effective) [182]
Lestauritinib	TRK, FLT3, JAK2	Phase I Study Well tolerated in patients with refractory neuroblastoma; effective and RP2D was established (120 mg/M(2)/dose BID) [183]	Phase III Study for the treatment of FLT3-ITD AML [184] No beneficial results shown
Gefitinib	EGFR	Phase II Study No significant results shown [185]	Approved by FDA for the treatment of patients with locally advanced or metastatic NSCLC [186]
Tivantinib	MET, EGFR, PDGFR-A, FGFR1/4	Phase I Study One NB patient had stable disease as best response RP2D is 240 mg/m^2^/dose, ORR not observed in this study [187]	Phase III Study for the treatment of adult patients with MET-high HCC No significant efficacy has been shown [188]
Cabozantinib	RET, c-MET, AXL, VEGFR2, FLT3, c-KIT	Case report: four NB patients 2/4 stable response, 2/4 stable disease; initiated doses ranged from 20 to 40 mg/m^2^/day [189]	Approved by FDA for the treatment of thyroid cancer, renal cell cancer, HCC and advanced neuroendocrine tumours [14], [https://www.targetedonc.com/view/fda-approves-cabozantinib-for-advanced-neuroendocrine-tumors (accessed on 10 October 2025)]
Sorafenib	VEGFR2-3, PDGFR-B, FLT3, c-KIT, RAF-1	Case report: four NB patients 3/4 no effectiveness shown, 1/4 stable tumour size for first 4 weeks; dose escalation to 250 mg/m^2^ [190] Phase II PEDS-PLAN Trial Recruiting at the moment (NCT02559778)	Approved by FDA to treat RCC and advanced HCC [191,192]
Regorafenib	VEGFR, PDGFR-B, c-KIT, RET, RAF-1	Phase I Study Efficacy shown in combination with vincristine and irinotecan with appropriate dose modifications; regorafenib RP2D is 82 mg/m^2^, ORR is 48% [193,194]	Approved by FDA for the treatment of HCC, GIST and metastatic colorectal cancer [https://www.fda.gov/drugs/resources-information-approved-drugs/regorafenib] [195,196]
Pazopanib	VEGFR1-3, PDGFR, FGFR1-2, c-KIT, CSF1R	Phase II Study No beneficial results shown pazopanib administered at 450 or 225 mg/m^2^ (NCT01956669)	Approved by FDA for the treatment of soft-tissue sarcoma and advanced RCC [197,198]

**Table 3:** RTK inhibitors and their application in neuroblastoma in ongoing phase I trials or preclinical research and other types of cancer. The table describes drug targets, research progress in NB with relevant clinical trial numbers where applicable, and research progress in other cancers.

Drug	Target	Stage of Research in Neuroblastoma	Stage of Research in Other Types of Cancer
Entrectinib	TRK, ALK, ROS1	Phase I Study Trial is active (NCT0265040)	Approved for the treatment of *NTRK* gene fusion solid tumours [199]
AZD6918	TRK	CDX (in combination with etoposide) [200]	Phase I Study for the treatment of adult patients with refractory solid malignancies Terminated (NCT00733031)
Repotrectinib	TRK fusion, ALK, ROS1	CDX [201]	Approved by FDA for the treatment of adult patients with locally advanced or metastatic ROS1-positive NSCLC [202]
Dacomitinib	EGFR, HER2, HER4	No data	Phase III Study for the treatment of patients with EGFR-mutated NSCLC (ARCHER 1050) More effective than crizotinib [203]
Osimertinib	EGFR	No data	Phase III Study for the treatment of patients with EGFR-mutated advanced NSCLC More effective than gefitinib or erlotinib [204]
Fexagratinib	FGFR1-3, VEGFR2	NB cell lines [109]	Phase I/II study for the treatment of FGFR fusion-positive glioma patients Has been beneficial, although additional investigation is required [205]
Erdafitinib	FGFR1-3, RET, CSF1R, PDGFR, FLT4, c-KIT, VEGFR2	PDX [107]	Approved by FDA to treat locally advanced or metastatic urothelial carcinoma [https://www.fda.gov/drugs/resources-information-approved-drugs/fda-approves-erdafitinib-locally-advanced-or-metastatic-urothelial-carcinoma]
Linsitinib	IGF-IR	NB cell lines [113]	Phase III Study for the treatment of patients with locally advanced or metastatic adrenocortical carcinoma No increase in overall survival shown [206]
AZD3463	IGF-IR, ALK	NB cell lines [207]	Breast cancer and glioblastoma cell lines [208,209]
Selpercatinib	RET	No data	Phase III Study for the treatment of patients with RET fusion-positive NSCLC Significantly prolonged progression-free survival [210]
Pralsetinib	RET	No data	Phase III ARROW Study for the treatment of patients with RET fusion-positive solid tumours Well tolerated treatment observed [211]
R-getretinib	RET	NB cell lines [212]	No data
Tepotinib	MET, TRKA, AXL, MER	CDX [213]	Approved by FDA to treat NSCLC harbouring MET alterations [214]
PHA665752	MET	NB cell lines [153]	Lung cancer CDX [215]
Bemcentinib	AXL, MER, TYRO3	NB cell lines [157,216]	Phase Ib/IIb Study for the treatment of patients with AML Bemcentinib alone and plus cytarabine are safe and well-tolerated [217]
Dubermatinib	AXL, TYRO3, MER, Aurora A, JAK2, ALK, ABL1, VEGFR2	CDX [218]	Phase I Study for the treatment of patients with advanced solid tumours Well tolerated treatment observed [219]
Sunitinib	PDGFR-B, VEGFR2, FLT3, c-KIT	CDX [220]	Approved by FDA for the treatment of renal cell cancer, GIST and pancreatic neuroendocrine tumour [14]
Axitinib	VEGFR1-3, PDGFR, c-KIT	Mice xenografts [221]	Approved by FDA for the treatment of RCC [14]

## Data Availability

No new data was generated in this manuscript.

## Supplementary Materials

The following supporting information can be downloaded at: https://www.mdpi.com/article/10.3390/cells15010004/s1. Table S1. Selectivity scores for RTK inhibitors from PRISM database and their targets depletion in neuroblastoma from DepMap. Table contains z-score for median values across NB cell lines compare to median values for all other tumour types, mean log fold change or gene scores for NB cell lines (NB_mean), mean log fold change or gene scores for all other cell lines (All_means), and *p*-value of NB vs all other cell lines comparison using Mann-Whitney non-parametric test.
