# Supplementary material for: Advances in Targeting Growth Factor Signalling in Neuroblastoma and Overcoming Drug Resistance
Source: Cells. 2025 Dec 19;15(1):4. doi: 10.3390/cells15010004 (PMC12785096; doi:10.3390/cells15010004)
Supplement: Supplementary file 1 [file cells-15-00004-s001.zip › cells-4005576-supplementary.pdf]

Gene	zscore	NB_mean	ALL_mean	vs_ALL_pvalue
ALK	-2.76275	-0.13773	-0.00672	8.35E-05
PIM1	-2.37398	-0.24466	-0.08791	8.19E-08
BCL2	-2.2272	-0.17296	-0.00867	6.41E-07
IGF1R	-1.64608	-0.46003	-0.23221	7.03E-06
KIT	-1.53764	-0.17293	-0.0747	1.75E-07
RET	-0.25384	-0.10607	-0.11323	0.484121
NTRK1	0.117865	-0.01407	-0.02248	0.401322
ERBB4	0.126809	-0.03764	-0.04863	0.488983
NTRK2	0.385497	0.083404	0.057622	0.186501
EGFR	0.463073	-0.14672	-0.24441	0.317471
ERBB2	0.766491	-0.13797	-0.2684	9.64E-05
AXL	1.052323	0.039658	-0.01411	0.018224
